# Functional Recovery Following Post-vaccination Neurological Syndrome With Demyelinating Findings on Magnetic Resonance Imaging (MRI): A Case Report

**DOI:** 10.7759/cureus.112004

**Published:** 2026-07-03

**Authors:** Hisham Hobbi, Mohammad Al-Jumaily, Hisham Al Hakim

**Affiliations:** 1 Medicine, Damascus University, Damascus, SYR; 2 Neurosurgery, American Spine Center, Dubai, ARE; 3 Neurology, American Spine Center, Dubai, ARE

**Keywords:** bnt162b2, case report, demyelination, exosome, goldic, hbot, neurorehabilitation, pfizer-biontech, vaccine-associated neurological syndrome

## Abstract

Rare neurological syndromes have been reported following severe acute respiratory syndrome coronavirus 2 (SARS-CoV-2) vaccination, although a causal relationship remains uncertain. We report the case of a 62-year-old woman who developed progressive neurological impairment temporally associated with BNT162b2 (Pfizer-BioNTech) COVID-19 vaccination, resulting in wheelchair dependence. Brain magnetic resonance imaging (MRI) demonstrated multiple periventricular T2-weighted/fluid-attenuated inversion recovery (T2/FLAIR) hyperintense lesions, including lesions oriented perpendicular to the lateral ventricles, consistent with demyelinating features. A multimodal therapeutic approach was implemented, including gut-directed therapy, intravenous gold-induced cytokine (GOLDIC®) therapy, hyperbaric oxygen therapy (HBOT), intranasal exosome therapy, and structured neurorehabilitation.

Over the course of treatment, the patient showed substantial clinical improvement, including recovery of ambulation and functional neurological status. While this case highlights the potential for significant neurological involvement following SARS-CoV-2 vaccination and the possibility of recovery with a multimodal therapeutic approach, the contribution of the individual therapies cannot be determined. Further research is needed to better understand the underlying mechanisms and to evaluate emerging therapeutic strategies, including GOLDIC, in this setting.

## Introduction

Post-vaccination syndrome is increasingly recognized as a complex multisystem condition characterized by persistent symptoms that may include severe immune-mediated neurological complications [1]. One of its rarest reported manifestations is the acute onset of demyelinating syndromes following BNT162b2 (Pfizer-BioNTech) mRNA vaccination [2]. Previous studies have also described rare immune-mediated central nervous system demyelinating disorders following COVID-19 vaccination, highlighting that such neurological complications, although uncommon, have been reported [3,4].

Demyelination refers to damage to the myelin sheath surrounding nerve fibers, resulting in impaired nerve signal conduction. Clinically, this may manifest as motor weakness, sensory disturbances, visual impairment, or autonomic dysfunction, depending on the affected regions of the central nervous system.

The proposed pathophysiology involves immune-mediated neuroinflammation, in which aberrant immune activation and molecular mimicry (a process in which immune responses may cross-react with self-antigens) may trigger inflammatory damage to myelin in susceptible individuals [5].

Management of post-vaccination neurological adverse events is based on established treatment protocols for analogous neuroimmunological conditions, as no vaccine-specific guidelines are currently available [6]. We present the case of a 62-year-old woman who developed acute demyelination following BNT162b2 vaccination, with symptom onset occurring shortly after the second dose. Her symptoms were managed using a tailored multimodal regimen combining regenerative therapies, specifically exosomes and GOLDIC, with hyperbaric oxygen therapy (HBOT).

## Case presentation

A 62-year-old woman presented with progressive worsening of limb weakness and stiffness. Her symptoms began after receiving two doses of the BNT162b2 (Pfizer-BioNTech) mRNA vaccine and were characterized by persistent stabbing pain in the lower mid-back (rated 8/10 on the Visual Analog Scale (VAS)), changes in speech, and progressive dysphagia. At the time of consultation, the patient was completely wheelchair-dependent. She had no significant past medical history and was not taking any regular medications before symptom onset.

Physical examination revealed bilateral positive Hoffmann's sign and a significant reduction in muscle strength, graded as 3/5 in both the upper and lower extremities. Hyperreflexia was present in both lower extremities, indicating upper motor neuron involvement. The clinical presentation was further characterized by bulbar symptoms, including dysphagia and dysphonia (nasal speech), and a sensory level extending to the C3-C4 dermatomes.

Contrast-enhanced brain magnetic resonance imaging (MRI) with axial T2-weighted/fluid-attenuated inversion recovery (T2/FLAIR) sequences (Figure 1) demonstrated multiple focal hyperintense lesions predominantly involving the bilateral periventricular white matter. The lesions were most prominent along the callososeptal interface and the ependymal surface of the corpus callosum. Several lesions were oriented perpendicular to the lateral ventricles, consistent with Dawson's finger-like morphology, a radiological feature commonly associated with demyelinating diseases such as multiple sclerosis that reflects perivenular inflammatory lesion spread. Additional small scattered hyperintense foci were observed in the bilateral frontoparietal white matter. No definite abnormal post-contrast enhancement was identified on the available images.

**Figure 1:**
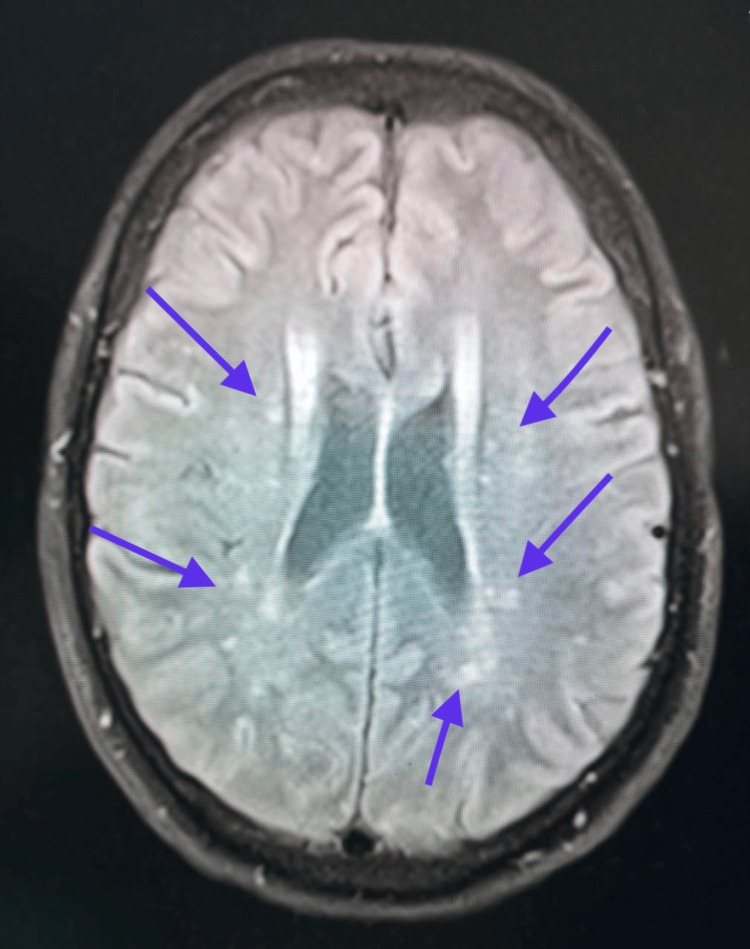
Axial T2/FLAIR MRI of the brain MRI of the brain showing multiple focal T2/FLAIR hyperintense lesions. The purple arrows indicate the characteristic periventricular lesions (Dawson’s fingers) showing a perpendicular orientation to the lateral ventricles. T2/FLAIR: T2-weighted/fluid-attenuated inversion recovery, MRI: magnetic resonance imaging.

A diagnostic lumbar puncture was performed. Cerebrospinal fluid (CSF) protein (32.2 mg/dL; reference range: 15-45 mg/dL) and glucose (63.4 mg/dL; reference range: 45-80 mg/dL) levels were within the normal range. No oligoclonal bands (OCBs) were detected in either the CSF or serum. Biochemical and hematological blood test results were within normal limits.

The patient was treated according to our institutional protocol. This included antibiotics (metronidazole 500 mg three times daily for two weeks, followed by once daily for six weeks) and the probiotic *Bifidobacterium lactis* throughout the course of antibiotic therapy for microbiota dysbiosis. GOLDIC® was administered via intravenous (IV) infusion in a total of eight sessions at three-day intervals according to the institutional protocol. Preparation of the autologous conditioned serum was performed according to established Orthokine/Autologous Conditioned Serum protocols previously described in the literature [6]. HBOT was administered at two atmospheres absolute (2 ATA) for one 60-minute session every three days, for a total of 10 sessions. Intranasal administration of 100 billion exosomes was performed once every 15 days for a total of two sessions. The treatment plan also included daily intensive neurorehabilitation (120-minute sessions for 30 days) and IV infusion of thiogamma (alpha-lipoic acid) 600 mg over 40 minutes, administered every other day for a total of seven sessions.

Outcome

Following completion of the multimodal regenerative treatment protocol, the patient demonstrated remarkable and sustained clinical recovery. Most notably, her mobility improved significantly. Previously completely wheelchair-dependent (Figure 2), she progressed to ambulating with minimal assistance (Figure 3). 

**Figure 2:**
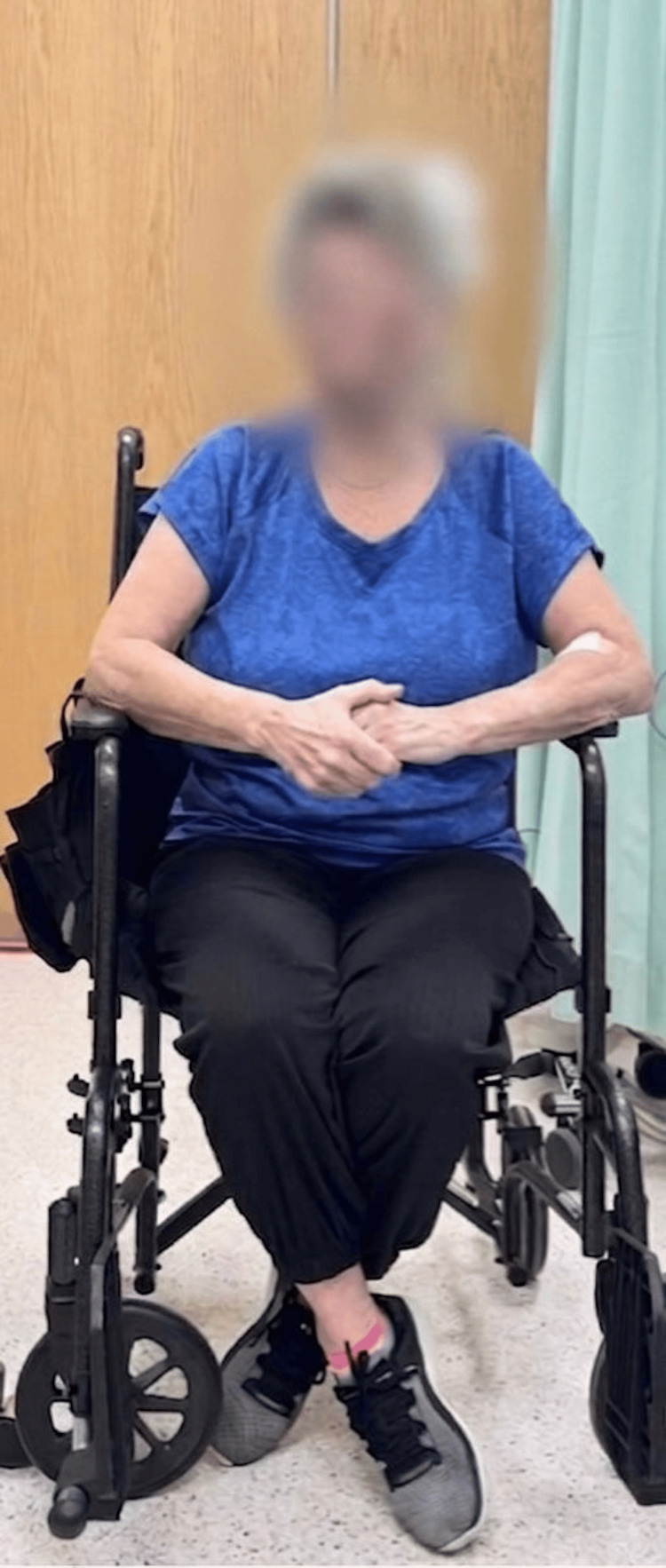
Patient's baseline mobility and neurological status prior to the therapeutic protocol

**Figure 3:**
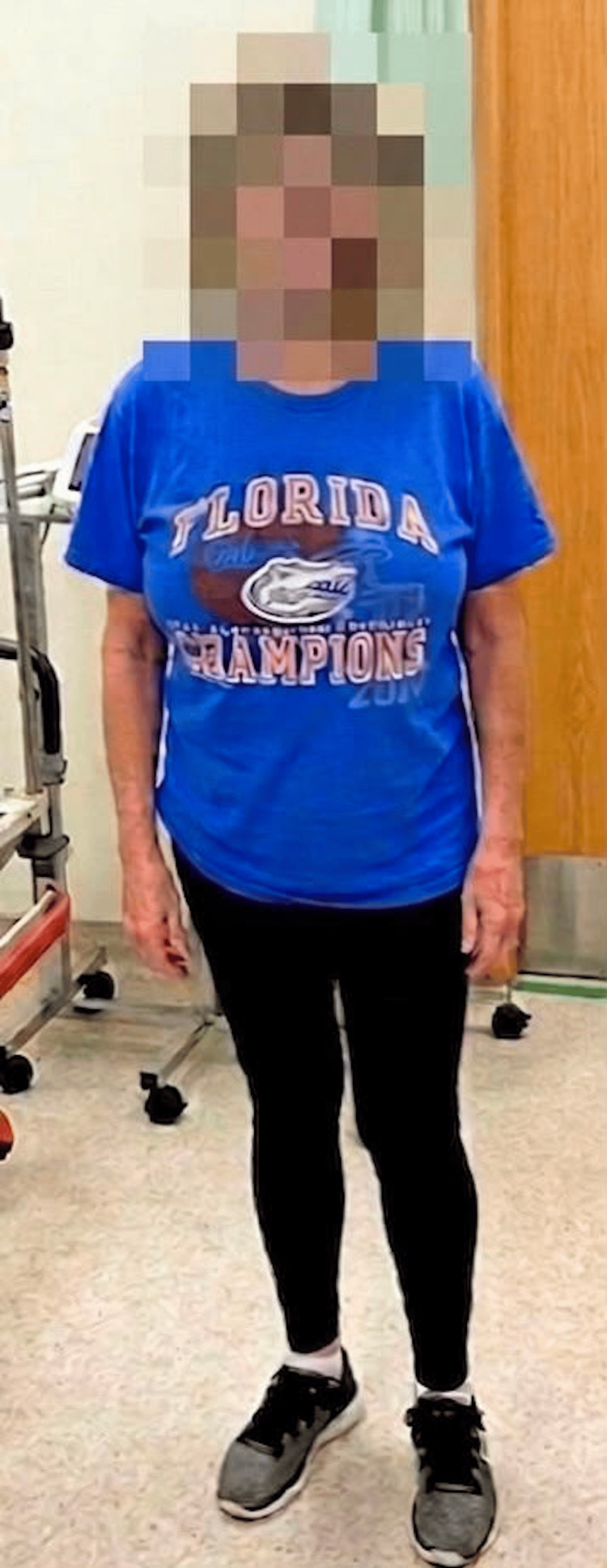
Significant clinical recovery and independent ambulation following the intervention

Neurological assessment demonstrated near-complete resolution of UMN signs. The previously debilitating muscle spasms resolved, and the hyperactive reflexes, which were prominent during the initial examination, returned to normal.

The patient's bulbar symptoms also improved markedly. Both dysphagia and speech clarity improved significantly, leading to a substantial enhancement in overall quality of life and daily functioning.

Furthermore, the stabbing pain in the mid-back that had significantly impaired her daily functioning, initially rated as 8/10 on the VAS, improved to a manageable 2/10. These combined findings represent substantial clinical improvement, reflecting the potential benefit of the multimodal neuroregenerative approach employed in this case.

The patient has been followed since completing treatment. During the follow-up period, no recurrence, complications, or new symptoms were observed, and she remained clinically stable.

The patient tolerated the multimodal treatment regimen, including exosome therapy, GOLDIC, and HBOT, without any treatment-related adverse events during the observation period. Written informed consent was obtained from the patient before treatment and for the publication of this case report.

## Discussion

A range of acute and persistent symptoms has been reported following COVID-19 vaccination in rare cases, involving multiple organ systems, although causality remains unproven and the underlying mechanisms are still under investigation [7]. Because the clinical presentation varies considerably among patients, we adopted a multimodal treatment strategy.

Our protocol initially included modulation of the gut-brain-immune axis. Dysbiosis and circulating inflammatory mediators such as lipopolysaccharides (LPS) may activate innate immune pathways, including Toll-like receptor 4 (TLR4), thereby contributing to microglial activation and persistent neuroinflammatory signaling [8].

Other factors, including host susceptibility, metabolic status, diet, and lifestyle, may also contribute to inflammatory responses and influence recovery.

The patient received GOLDIC® (gold-induced cytokine) therapy as an autologous serum-based intervention intended to modulate systemic inflammatory activity. The preparation involves ex vivo incubation of autologous blood under standardized conditions to induce cytokine release, following established Orthokine/Autologous Conditioned Serum protocols as described by Wehling et al. [6]. Preclinical and clinical reports suggest potential immunomodulatory effects with a favorable safety profile, although high-level evidence remains limited. Proposed effects include modulation of cytokine profiles and circulating immune mediators, although clinical evidence in demyelinating conditions remains limited [9]. GOLDIC therapy has been associated with modulation of circulating inflammatory mediators, including reported increases in gelsolin levels in synovial and peripheral blood compartments [9]. Experimental data suggest that gelsolin participates in the clearance of extracellular actin and may interact with inflammatory ligands such as LPS, potentially influencing innate immune signaling pathways [10]. In addition, autologous serum preparations may contain anti-inflammatory mediators such as interleukin-1 receptor antagonist (IL-1Ra) and granulocyte colony-stimulating factor (G-CSF), which are involved in the modulation of inflammatory signaling and immune cell responses [11]. Although these mechanisms remain largely hypothetical in the context of neurological disease, they may provide a possible biological explanation for the observed clinical course.

Intranasal exosome therapy was included to support neuroregenerative signaling. Preclinical studies suggest potential effects on oligodendrocyte function and remyelination pathways, with intranasal delivery offering a possible route for central nervous system targeting [12]. However, clinical evidence in humans remains limited. Adjunctive therapies included HBOT administered at 2 atmospheres absolute (2 ATA) to enhance tissue oxygenation [13] and alpha-lipoic acid to support mitochondrial function, reduce oxidative stress, and alleviate neurometabolic exhaustion [14]. Both have been explored in demyelinating and post-viral conditions, although the supporting evidence remains variable. A major contributor to the observed clinical improvement was likely the intensive neurorehabilitation program. Repetitive, task-specific training is known to promote activity-dependent neuroplasticity and cortical reorganization, which can support functional recovery even in the presence of structural central nervous system injury [15]. Importantly, given the multimodal nature of the treatment protocol, the individual contribution of each intervention cannot be determined. The observed clinical improvement may reflect the combined or synergistic effects of multiple therapies rather than the impact of any single intervention.

## Conclusions

This case highlights the complexity of neurological manifestations following SARS-CoV-2 vaccination and the potential involvement of inflammatory and demyelinating processes. Clinical improvement was observed in a patient with severe neurological impairment following a multimodal therapeutic approach. However, given the combined nature of the interventions, a causal relationship between the treatment and the observed improvement cannot be established.

These findings underscore the need for further investigation into the mechanisms underlying post-vaccination neurological complications and the potential role of emerging immunomodulatory and regenerative therapies, including GOLDIC®, in well-designed controlled clinical studies.
